# Supplementary material for: Any1 is a phospholipid scramblase involved in endosome biogenesis
Source: J Cell Biol. 2025 Mar 6;224(4):e202410013. doi: 10.1083/jcb.202410013 (PMC11893163; doi:10.1083/jcb.202410013)
Supplement: Table S1 — shows yeast strains used in this study (Gao et al.). [file jcb_202410013_tables1.docx]

**Table S1 – Yeast strains used in this study (Gao et al.)**

| **Strains** | **Genotype** | **Reference** |
| --- | --- | --- |
| BY4741 | MATa *his3∆1 leu2∆0 met15∆0 ura3∆0* | Euroscarf Library |
| SEY6210 | MATalpha *leu2-3 leu2-112 ura3-52 his3-∆200 trp1-∆101 lys2-801 suc2-∆9 GAL* | Gift of Reggiori Fulvio |
| SEY6210 MATa | MATalpha*::pSC11-MATa leu2-3,112 ura3-52 his3-∆200 trp-∆901 lys2-801 suc2-∆9 GAL* | Arlt et al., 2015 |
| CUY2232 | BY4741; *vps4∆::kanMX* | Euroscarf deletion library |
| CUY12940 | SEY6210; *MUP1-mGFP::hphNT1* | This study |
| CUY13158 | SEY6210; *MUP1-pHluorin::kanMX* | Gift of Scott Emr |
| CUY14302 | BY4741; *mCherry-CPS1::hphNT1* | This study |
| CUY14303 | BY4741; *vps4∆::kanMX mCherry-CPS1::hphNT1* | This study |
| CUY14616 | MATalpha *dga1∆::KanMX4 lro1∆::TRP1 are1∆::HIS3 are2∆::LEU2* | Gift of Florian Fröhlich |
| CUY14617 | MATalpha *dga1∆::KanMX4 lro1∆::TRP1 are1∆::HIS3 are2∆::LEU2 mCherry-Cps1::hphNT1* | This study |
| CUY14813 | BY4741; *vps4∆:: kanMX mCherry-CPS1::hphNT1 vps13∆::natNT2* | This study |
| CUY14814 | MATalpha *dga1∆::KanMX4 lro1∆::TRP1 are1∆::HIS3 are2∆::LEU2 mCherry-CPS1::hphNT1 vps4∆::URA* | This study |
| CUY14817 | BY4741; *mCherry-CPS1::hphNT1 vps13∆::natNT2* | This study |
| CUY14819 | BY4741; *vps4∆::kanMX mCherry-CPS1::hphNT1 SEC63-mNeon::HIS* | This study |
| CUY14939 | SEY6210; *vps13∆::kanMX MUP1-msGFP::natNT2* | This study |
| CUY14940 | SEY6210; *vps4∆::URA MUP1-msGFP::natNT2* | This study |
| CUY14941 | BY4741; *VPS13^GFP* | Gift of Arun Thomas John Peter; Lang et al., 2015 |
| CUY14942 | SEY6210; *MUP1-mGFP::hphNT1 ERG6-mKate::natNT2* | This study |
| CUY14946 | BY4741; *mCherry-CPS1::hphNT1 ANY1-mNeon::natNT2* | This study |
| CUY14947 | BY4741; *vps4∆::kanMX mCherry-CPS1::hphNT1*  *ANY1-mNeon::natNT2* | This study |
| CUY14948 | SEY6210; *VPS13-mGFP::natNT2 ANY1-mKate::hphNT1* | This study |
| CUY14949 | SEY6210; *vps4∆::URA VPS13-mGFP::natNT2*  *ANY1-mKate::hphNT1* | This study |
| CUY14950 | SEY6210; *MUP1-msGFP::natNT2* | This study |
| CUY14951 | SEY6210; *MUP1-mGFP::hphNT1 any1∆::natNT2* | This study |
| CUY14952 | BY4741; *vps4∆::kanMX mCherry-CPS1::hphNT1 any1∆::natNT2* | This study |
| CUY14953 | BY4741; *VPS13^GFP ANY1-mKate::hphNT1* | This study |
| CUY15041 | BY4741; *PHO5pr-Myc-GFP-YPT7::HIS ANY1-mKate::hphNT1* | This study |
| CUY15042 | BY4741; *PHO5pr-Myc-GFP-VPS21::HIS ANY1-mKate::hphNT1* | This study |
| CUY15046 | BY4741; *ANY1-mKate::hphNT1 SEC7-mNeon::HIS* | This study |
| CUY15047 | BY4741; *vps4∆::kanMX mCherry-CPS1::hphNT1 ypt35∆::HIS* | This study |
| CUY15049 | BY4741; *vps35∆::kanMX ANY1-mNeon::HIS* | This study |
| CUY15050 | BY4741; *GAL1pr-ANY1::kanMX FLAG::MET* | This study |
| CUY15212 | SEY6210; *VPS4-HA-mCherry::TRP GFP-YPT7::HIS* | This study |
| CUY15213 | SEY6210; *VPS4-HA-mCherry::TRP GFP-YPT7::HIS*  *any1∆::hphNT1* | This study |
| CUY15222 | BY4741; *mCherry-CPS1::hphNT1 SEC63-mNeon::HIS* | This study |
| CUY15266 | BY4741; *ANY1-Chromobody::hphNT1* | This study |
| CUY15267 | BY4741; *PHO5pr-Myc-GFP-YPT7::HIS*  *ANY1-Chromobody::hphNT1* | This study |
| CUY15268 | BY4741; *PHO5pr-Myc-GFP-VPS21::HIS*  *ANY1-Chromobody::hphNT1* | This study |
| CUY15269 | BY4741; *ANY1-Chromobody::hphNT1 SEC7-msGFP::natNT2* | This study |
| CUY15275 | BY4741; *vps5∆::natNT2 ANY1-mNeon::HIS* | This study |
| CUY15276 | BY4741; *vps18∆::kanMX ANY1-mNeon::HIS* | This study |
| CUY15400 | SEY6210; *MUP1-mGFP::hphNT1 ERG6-mKate::natNT2 vps13∆::kanMX* | This study |
| CUY15402 | SEY6210; *vps13∆::kanMX MUP1-msGFP::natNT2 SEC63-3xmCherry::hphNT1* | This study |
| CUY15404 | BY4741; *any1∆::hphNT1* | This study |
| CUY15406 | SEY6210; *MUP1-msGFP::natNT2 SEC63-3xmCherry::hphNT1* | This study |
| CUY15471 | SEY6210 MATa; *VPS13-mNeon::hphNT1*  *FAA4-mScarlet::kanMX* | This study |
| CUY15675 | SEY6210 MATa; *any1∆::hphNT1* | This study |
| CUY15676 | SEY6210; *MUP1-pHluorin::kanMX any1∆::hphNT1* | This study |
| CUY15677 | BY4741; *any1∆::hphNT1 mCherry-CPS1::URA* | This study |
| CUY15679 | SEY6210 MATa; *any1∆::hphNT1 pRS406-ANY1pr-ANY1-mNeon::URA* | This study |
| CUY15680 | SEY6210 MATa; *any1∆::hphNT1 pRS406-ANY1pr-ANY1(T60L S64L)-mNeon::URA* | This study |
| CUY15684 | SEY6210 MATa; *any1∆::hphNT1 pRS406-ANY1pr-ANY1-mNeon::URA SEC7-mScarlet::kanMX* | This study |
| CUY15685 | SEY6210 MATa; *any1∆::hphNT1 pRS406-ANY1pr-ANY1-mNeon::URA mCherry-YPT7::kanMX* | This study |
| CUY15686 | SEY6210 MATa; *any1∆::hphNT1 pRS406-ANY1pr-ANY1-mNeon::URA mCherry-VPS21::kanMX* | This study |
| CUY15687 | SEY6210 MATa; *any1∆::hphNT1 pRS406-ANY1pr-ANY1(T60L S64L)-mNeon::URA SEC7-mScarlet::kanMX* | This study |
| CUY15688 | SEY6210 MATa; *any1∆::hphNT1 pRS406-ANY1pr-ANY1(T60L S64L)-mNeon::URA mCherry-YPT7::kanMX* | This study |
| CUY15689 | SEY6210 MATa; *any1∆::hphNT1 pRS406-ANY1pr-ANY1(T60L S64L)-mNeon::URA mCherry-VPS21::kanMX* | This study |

**Reference:**

Arlt, H., K. Auffarth, R. Kurre, D. Lisse, J. Piehler, and C. Ungermann. 2015. Spatiotemporal dynamics of membrane remodeling and fusion proteins during endocytic transport. *Mol. Biol. Cell.* 26:1357–1370. doi: 10.1091/mbc.E14-08-1318

Lang A.B., A.T. John Peter, P. Walter, B. Kornmann. 2015. ER–mitochondrial junctions can be bypassed by dominant mutations in the endosomal protein Vps13. *J. Cell Biol*. 210 (6): 883–890. doi: 10.1083/jcb.201502105
