# Supplementary material for: Any1 is a phospholipid scramblase involved in endosome biogenesis
Source: J Cell Biol. 2025 Mar 6;224(4):e202410013. doi: 10.1083/jcb.202410013 (PMC11893163; doi:10.1083/jcb.202410013)
Supplement: Table S2 — shows plasmids used in this study (Gao et al.). [file jcb_202410013_tables2.docx]

**Table S2 – Plasmids used in this study (Gao et al.)**

| **Plasmids** | **Reference** |
| --- | --- |
| pRS406-*vps4ts* | Gift of Markus Babst |
| *pRS406-ANY1pr-ANY1-mNeon* | This study |
| *pRS406-ANY1pr-ANY1(T60L S64L)-mNeon* | This study |
