# Supplementary figures and images for: Any1 is a phospholipid scramblase involved in endosome biogenesis
Source: J Cell Biol. 2025 Mar 6;224(4):e202410013. doi: 10.1083/jcb.202410013 (PMC11893163; doi:10.1083/jcb.202410013)

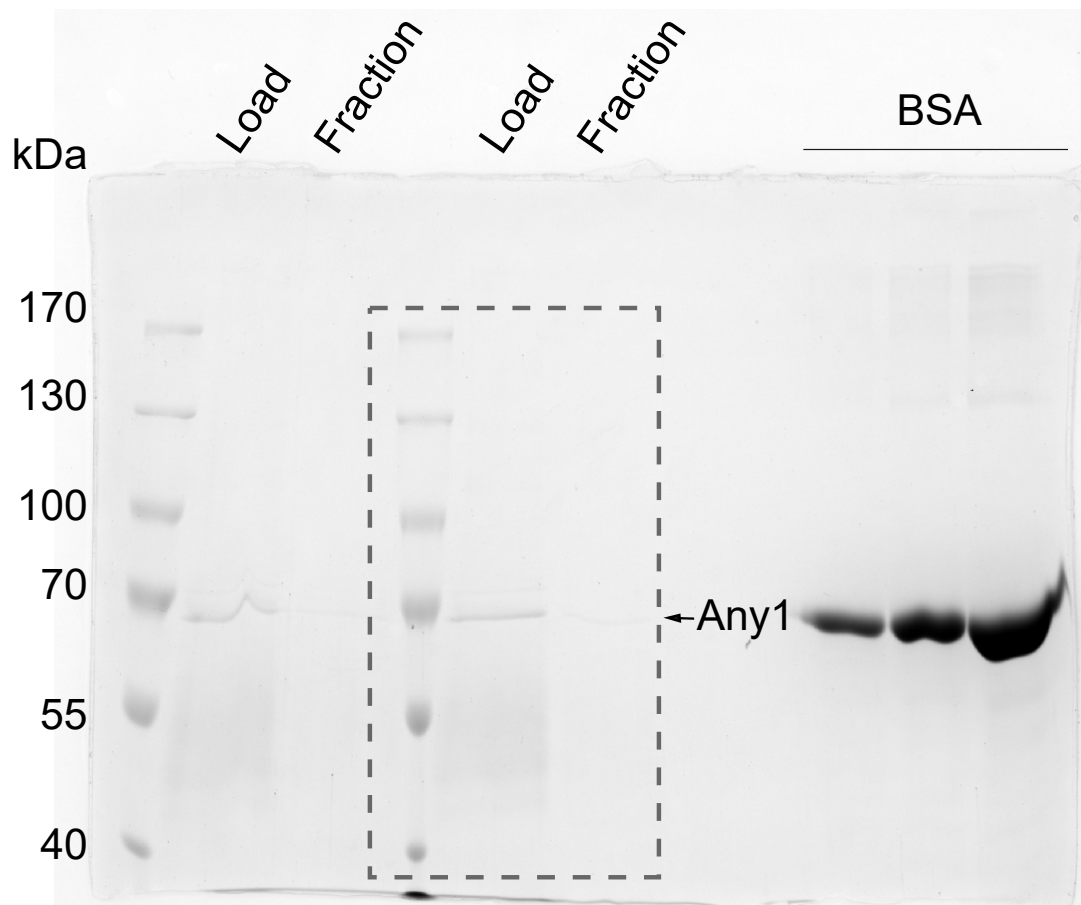

Supplement: SourceData FS2 — is the source file for Fig. S2. [file jcb_202410013_sourcedatafs2.pdf]
